# Ventilator-Associated Pneumonia in Neonates Admitted to a Tertiary Care NICU in Bulgaria

**DOI:** 10.3389/fped.2022.909217

**Published:** 2022-06-28

**Authors:** Vanya R. Rangelova, Ralitsa D. Raycheva, Ani K. Kevorkyan, Maya B. Krasteva, Yordan I. Kalchev

**Affiliations:** ^1^Department of Epidemiology and Disaster Medicine, Faculty of Public Health, Medical University of Plovdiv, Plovdiv, Bulgaria; ^2^Department of Social Medicine and Public Health, Faculty of Public Health, Medical University of Plovdiv, Plovdiv, Bulgaria; ^3^Department of Obstetrics and Gynecology, Neonatology Unit, Faculty of Medicine, Medical University of Plovdiv, Plovdiv, Bulgaria; ^4^Department of Microbiology and Immunology, Faculty of Pharmacy, Medical University of Plovdiv, Plovdiv, Bulgaria

**Keywords:** ventilator-associated pneumonia, healthcare-associated infection, neonatology intensive care unit, nosocomial infection, risk factors

## Abstract

Ventilator-associated pneumonia (VAP) is the second most common hospital-acquired infection (HAI) among neonatal patients in the intensive care units (ICUs) and is a serious challenge for neonatologists because it affects critically ill patients who need prolonged mechanical ventilation. In Bulgaria, there is no detailed data at regional and national levels on the characteristics of VAP in newborns, which imposes a necessity for specific studies of risk factors and etiology of VAP. The aim of the study was to analyze the frequency, characteristics and risk factors for the occurrence of VAP in newborns hospitalized in intensive care unit. This was a prospective study, conducted between January 2017 and June 2018 in the NICU of University Hospital “St. George” Plovdiv, Bulgaria. The sample consisted of 507 neonates, followed up prospectively, 107 of whom were placed on mechanical ventilation for ≥48 h. VAP was diagnosed in 33 out of 107 neonates (31%). The VAP incidence rate was 35.06/1.000 ventilator days. We confirmed differences between the median birth weight (1,310 vs. 1,690 g, *p* = 0.045) and average gestational age (31.08 g.w. vs. 33.08 g.w, *p* = 0.04) of the patients with and without VAP. The average stay of patients with VAP in the NICU was statistically significantly longer than the hospital stay of non-VAP patients (35.70 ± 21.84 days vs. 21.77 ± 17.27 days (*t* = 3.241, *p* = 0.002). In neonates with VAP, the duration of mechanical ventilation was statistically significantly longer compared with non-VAP patients (16.88 ± 11.99 vs. 5.42 ± 4.48; *t* = 5.249, *p* = 0.000). A statistically significant prevalence of Gram-negative bacteria among VAP patients was demonstrated (91%) compared to the Gram-positive (9%), *p* < 0.05. The leading causative agent of VAP was *Klebsiella pneumoniae* ESBLs + (27%), followed by *Acinetobacter baumannii* (14%), *Pseudomonas aeruginosa* (12%) and *Escherichia coli* (12%). In multivariate logistic regression, mechanical ventilation >7 days was established as an independent risk factor for VAP (OR 3.6; 95% CI: 1.7–6.5, *p* = 0.003). VAP remains a serious and outstanding issue in pediatric and neonatal intensive care units. The findings of the current study emphasize that the birth weight, gestational age, and duration of hospital stay have a significant association with ventilator-associated pneumonia.

## Introduction

Mechanical ventilation (MV) is an essential part of modern neonatal intensive care. However, it is associated with a substantial risk of ventilator-associated pneumonia (VAP) (1). VAP has a serious impact on neonatal morbidity and mortality and on treatment costs (2, 3). Ventilator-associated pneumonia is defined as a lung infection in mechanically ventilated patients, starting ≥48 h after the initiation of mechanical ventilation (4). The clinical criteria for diagnosing VAP have been developed by the Center for Disease Control (5) and further, the German national nosocomial infection surveillance system (Krankenhaus Infektions Surveillance System [KISS]) offers a definition for very low birth weight infants in their NEO-KISS module (6). However, it should be noted that there is no gold standard for the diagnosis of this infection in the neonatal period and differences are noticed in the applied criteria (7, 8). Additionally, neonates have different anatomy, physiology and underlying diseases and undergo different invasive procedures.

In 2012 the European Center for Disease Control and Prevention (ECDC) conducted the first point prevalence survey on nosocomial infections in Bulgaria, registered in all age groups and cases of pneumonia in newborns were estimated to be 2.3% (9). In the same period, Gladilova and Ribarova (10) discovered that 2% of all hospitalized neonates were diagnosed with a nosocomial infection as in this report, the information from special care baby units (SCBU) and NICUs was not separated. There is no detailed data at regional and national levels on the characteristics of VAP in newborns, which imposes a necessity for specific studies of risk factors and etiology of VAP.

The aim of the study was to analyze the frequency, characteristics and risk factors for the occurrence of VAP in newborns hospitalized in intensive care unit.

## Materials and Methods

### Study Period and Setting

A prospective cohort study was conducted at the level 3 NICU of University Hospital “St. George” Plovdiv, Bulgaria from January 2017 to June 2018. The hospital is located in the second largest city and provides complex clinical care to patients from the South-Central region (~20.0% of the overall Bulgarian population).

### Patient Characteristics

The sample consisted of 507 neonates, followed up prospectively, 107 of whom were placed on mechanical ventilation for ≥48 h. Data on demographic characteristics of those patients, underlying diseases, clinical symptoms, imaging studies, etiological agents isolated from clinical samples (endotracheal aspirates, blood cultures, ear secretions, wound samples), and antimicrobial susceptibility rates were recorded.

### Definition and Identification of VAP

We used the German system for surveillance of nosocomial infections NEO KISS (6) and the Centers for Disease Control and Prevention (CDC) (5) to define the criteria for VAP cases. Additionally, we applied criteria for VAP from the Bulgarian Medical Standard for Prevention and Control of Nosocomial Infections (11). The survey forms for epidemiological surveillance of VAP were prepared based on clinical, laboratory and radiological criteria after a landscape review of the literature. VAP was defined as pneumonia where the patient is on mechanical ventilation for > or ≥2 consecutive calendar days on the date of onset with at least two or more clinical and laboratory signs and symptoms, chest X-ray imaging showing new or persistent infiltrates, consolidation or pleural effusion and isolation of a pathogen from the endotracheal aspirate. The clinical signs included: elevated temperature >37.8°C, hypothermia, frequent apnea/bradypnea/tachypnea, bradycardia <80 b/m, change in tracheal secretions (color, quantity). Laboratory findings included: C-reactive protein test (CRP) >10 mg/L, abnormal white blood cells count (Leu >30,000/mcg or Leu <5,000/mcg), thrombocytopenia (Thr <150,000/μl). Patients had to fulfill clinical, radiological, and microbiological criteria to be diagnosed with ventilator-associated pneumonia.

### Microbiological Methods

Direct microscopy on the clinical samples was performed using 100 × immersion oil microscopy after staining the slides with Gram and methylene blue stain. The latter assists in the differentiation between colonization and infection, based on the presence of polymorphonuclear cells. Endotracheal specimens were cultured on blood-agar, eosin-methylene blue agar (Levin's medium) and thioglycolate broth. Semi-automated (API 20 NE, bioMerieux) and automated systems (Vitek-2 Compact, bioMerieux and MALDI-TOF MS, Vitek-MS, bioMerieux) were used for bacterial identification. Antimicrobial susceptibility testing was performed on Müller-Hinton agar by using the disk-diffusion method of Bauer-Kirby and by using *E*-test and broth microdilution test to determine the minimal inhibitory concentrations (MIC), as well as automated systems (Vitek-2 Compact, bioMerieux).

### Statistical Methods

Quantitative variables are presented as the mean ± standard deviation (mean ± SD) or median (25th percentile; 75th percentile) based on the sample distribution. The Kolmogorov-Smirnov test was applied to inform about the distribution of the patients sampled. The variables were compared for differences using independent samples *t*-test or Mann–Whitney test based on the normality of the distribution. Qualitative variables are presented as numbers/totals and percentages (*n*, %), and a *z*-test was applied to compare two proportions. Multivariate logistic regression analysis was used to find the independent risk factors associated with ventilator-associated pneumonia. The univariate analysis was applied to identify the variables with a *p*-value <0.10 to be introduced in the model. The *p*-values < 0.05 were considered statistically significant for all statistical tests. The systematization, processing, and analysis of the data were performed using SPSS v.26 for Windows (IBM 141 Corp. Released 2019. Armonk, NY: IBM Corp).

### Ethical Considerations

The study received approval from the Ethics Committee of the Medical University of Plovdiv, Bulgaria.

## Results

The total number of neonates admitted during the study is 507. One hundred and seven (21%) of the newborns required mechanical ventilation for ≥48 h and were enrolled in the current study. VAP was diagnosed in 33 neonates (31%). The VAP incidence rate was 35.06/1.000 ventilator days. During the study among the 107 studied neonates additionally six cases of bloodstream infection and three cases of conjunctivitis were diagnosed. The rest of the neonates (*n* = 74; 69%) were considered to be a control group for further comparisons with the exposed group. The patient characteristics of both VAP and non-VAP patients are presented in Table 1.

**Table 1:** Characteristics of the neonates with and without VAP.

**Characteristics**	**VAP** **(*n* = 33) *n* (%)**	**Non-VAP** **(*n* = 74) *n* (%)**	***p*-value**
Male gender	20 (67)	40 (54)	0.530
Mean birth weight in grams	1,310 (965; 2,400)	1,690 (1,207; 2,703)	0.040
(min: max)			
**>2,500 gr**.	7 (21)	22 (30)	
2,499–1,500 g.	5 (15)	22 (30)	
1,499–1,000 g.	12 (37)	18 (24)	
<1,000 g	9 (27)	12 (16)	
Age in gestational week, gw	31.08 ± 4.83	33.08 ± 4.33	0.045
(mean ± SD)			
**>37 g.w**.	6 (18)	18 (24)	
36–32 g.w.	5 (15)	27 (36)	
31–28 g.w.	9 (27)	15 (20)	
<28 g.w.	13 (40)	14 (18)	
**Mode of delivery**
Normal vaginal delivery (NVD)	11 (33)	18 (24)	(0.540)
Cesarean section (CS)	22 (67)	56 (76)	
Apgar score at 1 min	4.7 ± 2.6	5.0 ± 2.5	
Apgar score at 5 min	7.7 ± 2.6	8.1 ± 1.9	
Length of hospital stay	35.7 ± 21.84	21.77 ± 17.27	0.002
(patient days)			
Mechanical ventilation (days)	16.88 ± 11.99	5.42 ± 4.48	0.000
CVC^*^/UVC^**^ use (days)	8.5 ± 6.8	5.0 ± 3.6	0.007
PVC^***^ use (days)	35.1 ± 21.5	20.5 ± 16.3	0.001
Antibiotic treatment (days)	31.2 ± 19.5	18.4 ± 13.5	0.001
Number of antibiotics used	5.73 ± 1.96	3.64 ± 1.8	0.000

^*^CVC, central venous catheter; ^**^UVC, umbilical venous catheter; ^***^PVC, peripheral venous catheter.

The mean birth weight of the patients with VAP was significantly lower than the birth weight of non-VAP patients, there was an association between the low birth weight and VAP (χ^2^ = 7.437, *p* = 0.024). Moreover, we found an association between premature birth (<37 weeks of gestation) and VAP (χ^2^ = 10.02, *p* = 0.018).

For all 107 patients enrolled in the study 2,789 patient days were calculated. Of these, 1,178 (42%) were registered among the 33 neonates with VAP. The average stay of patients with VAP in the NICU was statistically significantly longer than the hospital stay of non-VAP patients (35.70 ± 21.84 days vs. 21.77 ± 17.27 days (*t* = 3.241, *p* = 0.002). Among the 107 newborns included in the study 941 days of mechanical ventilation were recorded. The device utilization ratio of mechanical ventilation was estimated at 33.74/100 patient days. In neonates with VAP, the duration of mechanical ventilation was statistically significantly longer compared with non-VAP patients (16.88 ± 11.99 vs. 5.42 ± 4.48; *t* = 5.249, *p* = 0.000). The mean estimated time to diagnosis of VAP was 8 ± 5 days. During the study period, three infants diagnosed with VAP died which resulted in a mortality rate of 9%.

In Table 2 the most common findings in patients with VAP are presented.

**Table 2:** Clinical symptoms, laboratory findings and X-ray findings of 33 infants with VAP.

**Clinical symptoms**	***n* (%)**
Increased temperature	4 (12)
Tachycardia	5 (56)
Change in the color of the respiratory secretions	31 (94)
Change in the quantity of respiratory secretions	21 (64)
**Laboratory findings**	
Elevated CRP	13 (40)
Leukocytosis/leukopenia	15 (46)
Thrombocytopenia	23 (70)
**Radiological findings**	
Data of pneumothorax or drainage of the pleural cavity	15 (46)
X-ray changes at admission in NICU	23 (70)
X-ray changes during the hospital stay	21 (64)

### Etiological Structure of VAP

In patients with VAP, a total of 66 microorganisms were isolated from tracheal aspirates, which are presented in Table 3.

**Table 3:** Microorganisms isolated from tracheal aspirates in patients with VAP (*n* = 66).

**Microorganism**	**Tracheal isolates** **(causative agents of VAP) *n* (%)**
**Gram positive**	
*CoNS (coagulase-negative staphylococci)*	1 (1.5)
*Enterococcus faecalis*	4 (6)
*Enterococcus faecium*	1 (1.5)
**Overall gram positive**, ***n***	6 (9)
**Gram negative**	
*Klebsiella pneumoniae ESBL+*	18 (27)
*Klebsiella oxytoca ESBL+*	4 (6)
*Escherichia coli*	8 (12)
*Enterobacter cloacae compelex ESBLs+*	2 (3)
*Pantoea agglomerans (Enterobacter agglomerans)*	1 (2)
*Pseudomonas aeruginosa*	8(12)
*Acinetobacter baumannii*	9 (14)
*Acinetobacter lwoffii*	1 (2)
*Strenotrophomonas maltophilia*	4 (6)
*Chryseobacterium indologenes*	2 (3)
*Chryseobacterium gleum*	1 (1)
*Achromobacter xylosoxidans*	2 (3)
**Overall gram negative**, ***n***	60 (91)
**Overall isolates**, ***n***	66

A statistically significant prevalence of Gram-negative bacteria was demonstrated (91%) compared to the Gram-positive (9%), *p* < 0.05. The leading causative agent of VAP was *Klebsiella pneumoniae ESBLs*+ (27%), followed by *Acinetobacter baumannii* (14%), *Pseudomonas aeruginosa* (12%) and *Escherichia coli* (12%). These four microorganisms accounted for 65% of the agents isolated in VAP patients.

**Figure 1:**
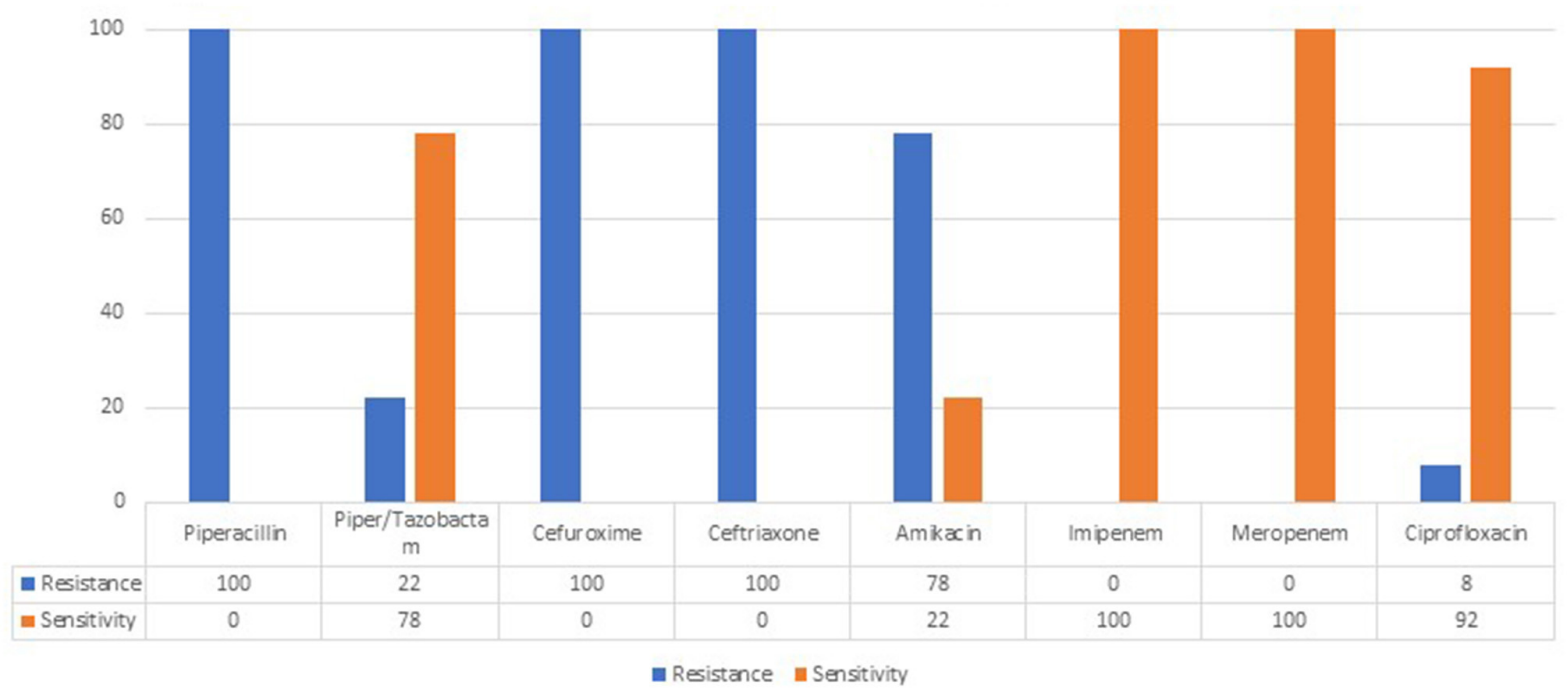
Antibiotic resistance of *Klebsiella pneumoniae* ESBL+.

Isolated strains of the leading pathogen *Klebsiella pneumoniae ESBLs*+ showed 100% resistance to second-and third-generation cephalosporins. No carbapenemase-producing *K. pneumoniae* strains were isolated (Figure 1).

Table 4 summarizes the results of univariate analyses of potential factors that were associated with VAP manifestation in this cohort. All significant variables from the univariate statistics were included in the multivariate analysis.

**Table 4:** Univariate analysis of the risk factors associated with the VAP manifestation.

**Variable**	**VAP patients (*n* = 33)**	**Non-VAP patients (*n* = 74)**	**OR^**a**^**	**95%CI^**b**^**	***p*-value^**c**^**
**Birth weight (<1,499 gr.)**	21	30	3.08	1.00–9.44	0.043
**Gestational age (<32 gw)**	22	29	4.10	1.40–12.35	0.009
**Resuscitation**	29	62	1.40	0.42–4.73	0.583
**Intubation**	25	47	1.80	0.71–4.53	0.212
**Patient days (>7 days)**	33	60	1.55	1.33–1.80	0.007
**Patient days (>14 days)**	27	47	2.59	0.95–7.05	0.058
**Duration of use of CVC/UVC (>7 days)**	13	8	5.28	1.92–14.5	<0.001
**Duration of use of PVC (>7 days)**	33	60	1.55	1.33–1.80	0.007
**Duration of MV (>7 days)**	25	19	9.05	3.49–23.43	<0.0001
**Duration of MV (>14 days)**	6	4	3.89	1.02–14.86	0.036
**Duration of AB therapy (>7 days)**	32	60	7.47	0.94–59.40	0.029
**Duration of AB therapy (>14 days)**	26	39	3.33	1.29–8.63	0.011

^a^*OR, odds ratio; ^b^95% CI, 95% confidence interval*.

*^c^Pearson χ^2^*.

Multivariate logistic regression model was statistically significant (χ^2^ = 36.49, *df* = 13; *p* < 0.001). The model explained 51.9% (Nagelkerke R2) of the variations in VAP and correctly classified 87.3%. In the case of multivariate logistic regression, mechanical ventilation >7 days (OR 3.6; 95% CI: 1.7–6.5, *p* = 0.003) was established as an independent risk factor for VAP. The duration of MV was defined as the duration of MV before the diagnosis of VAP.

## Discussion

Ventilator-associated pneumonia is one of the most common healthcare-associated infections in the critical care setting (12). Previous studies, evaluating the incidence rate of VAP in NICU patients, observed inconsistent results varying from 8.1 to 57.1% (13–15). Furthermore, the reported incidence rate depends on the type of hospital and the economic development of the country, where the study was conducted (16).

Much of the current literature on common risk factors for VAP pays particular attention to low birth weight and premature birth (13, 17). Our results corroborate these findings. Moreover, 64% of all neonates with VAP were born with a birth weight <1,499 g. In accordance with the present results, previous studies by Chastre et al. (18) and Stover et al. (19) have reported that VAP rates were highest for the 1–1.499 kg birth weight categories.

The mortality among patients with VAP was estimated at 9% which corresponds with other studies (14, 20).

The hospital stay of patients with VAP was statistically significantly longer compared to the non-VAP patients. Similar are the results for the duration of MV in both groups. A comparison of the findings with those of other studies confirms the statistically significant difference in the MV duration between VAP and non-VAP patients (13, 21, 22). Prolonged mechanical ventilation and its connection to VAP can also be explained by an increased risk of infection due to exposure to nebulizers, humidifiers and more changes in the respiratory circuits that are known to be important sources and a breeding ground for pathogenic microorganisms (22).

Regarding the clinical symptoms in patients with VAP, the change in the color and quantity of the respiratory secretions were most reported, which is in accordance with other studies (14, 15). The most common deviation in the laboratory findings in patients with VAP was thrombocytopenia - 70% (*n* = 23) followed by leukocytosis/leukopenia (46%, *n* = 15). In the literature, thrombocytopenia and leukocytosis/leukopenia are also discussed as hematological changes characteristic of nosocomial infections, including VAP (23). However, it should be taken into consideration that leukopenia/leukocytosis may also be reported in other conditions (newborns born to mothers with preeclampsia (24), steroid treatment, due to bronchopulmonary dysplasia (25) and the neonatologist's assessment of the child's condition and the relationship of hematological changes to the diagnosis of VAP is extremely important.

X-ray changes were observed in all of the patients with VAP as this was one of the criteria to make a VAP diagnosis. Badr et al. (26) and El-ward et al. (27) also found a very high proportion of patients with VAP in whom a change in a radiographic image has been reported.

Considering the microbiology of VAP a statistically significant prevalence of Gram-negative bacteria was demonstrated (91%) compared to the Gram-positive (9%), *p* < 0.05. A large and growing body of literature demonstrates that Gram-negative microorganisms have been the most frequent causative agents of VAP (7, 13, 22, 26). Conversely, in some reports, Gram-positive microorganisms were more commonly isolated: mainly *Staphylococcus aureus* (28). Epidemiological assessment of the prevalence of Gram-negative microorganisms in VAP is important when planning diagnostic and intervention strategies.

The most frequently reported agent was *K. pneumonia ESBLs*+ (27%), followed by *A. baumannii* (14%), *P. aeruginosa* (12%) and *E. coli* (12%). These findings correspond to a previous retrospective study conducted in the NICU (29) with respect to the etiological risk factors of VAP, as well as research by other authors (14, 18).

The isolated strains of *K. pneumoniae ESBLs*+ demonstrated 100% resistance to second-and third-generation cephalosporins. This finding broadly supports the work of other studies in this area, where the change in the characteristics of this microorganism is widely discussed (30). The comparison between a previous retrospective study (29) done in the same unit and our results indicate increased isolation of broad-spectrum beta-lactamase-producing *K. pneumoniae*.

In the case of binary logistic regression mechanical ventilation >7 days was established as an independent risk factor for VAP (OR 3.6; 95% CI: 1.7–6.5, *p* = 0.003). Other similar studies have also reported mechanical ventilation as an independent significant risk factor for VAP in neonates (17, 31).

### Limitations of the Study

Our research has several limitations, including the lack of matched cases in our case-control study. However, speaking about epidemiological case-control studies there are records in the literature in which the same or even identical results were found irrespective of whether matching or not matching was applied (32). In addition, this study was conducted in one hospital, but it is located in the second largest Bulgarian city and moreover, the NICU of this hospital is the only available option for the South-Central Region population, which represents ~20.0% of the overall Bulgarian population (33). We could consider that “St. George” NICU's resources, staff and patient numbers are similar to the NICUs located in the other five Bulgarian regions. Ideally, sampling across other NICUs in Bulgaria would have supported the case for the generalizability of the findings. No attempt has been made to measure the impact of functional deficits in patients with VAP.

## Conclusion

Ventilator-associated pneumonia remains a serious and outstanding issue in pediatric and neonatal intensive care units. The findings of the current study emphasize that the birth weight, gestational age, and duration of hospital stay have a significant association with ventilator-associated pneumonia. Prevention and timely treatment of this nosocomial infection is critical for improving the prognosis of these neonates.

## Data Availability Statement

The raw data supporting the conclusions of this article will be made available by the authors, without undue reservation.

## Ethics Statement

The studies involving human participants were reviewed and approved by Ethics Committee of Medical University of Plovdiv, Bulgaria. Written informed consent to participate in this study was provided by the participants' legal guardian/next of kin.

## Author Contributions

VR is the main author, who wrote the manuscript and provided the patients' information and created the initial database. RR is responsible for the statistics and editing of the manuscript. YK is responsible for the microbiological interpretation and editing of the manuscript. AK and MK are the supervisors responsible for the editing and final corrections. All authors contributed to the article and approved the submitted version.

## Conflict of Interest

The authors declare that the research was conducted in the absence of any commercial or financial relationships that could be construed as a potential conflict of interest.

## Publisher's Note

All claims expressed in this article are solely those of the authors and do not necessarily represent those of their affiliated organizations, or those of the publisher, the editors and the reviewers. Any product that may be evaluated in this article, or claim that may be made by its manufacturer, is not guaranteed or endorsed by the publisher.

## References

[1] Aly H Badawy M El-Kholy A Nabil R. Mohamed A. Randomized, controlled trial on tracheal colonization of ventilated infants: can gravity prevent ventilator-associated pneumonia? Pediatrics. (2008) 122:770–4. 10.1542/peds.2007-1826 18829800

[2] Gaynes RP Edwards JR Jarvis WR Culver DH Tolson JS Martone WJ. Nosocomial infections among neonates in high-risk nurseries in the United States. National Nosocomial Infections Surveillance System. Pediatrics. (1996) 98:357–61. 10.1542/peds.98.3.357 8784356

[3] Stoll BJ Hansen NI Adams-Chapman I Fanaroff AA Hintz SR Vohr B. National Institute of Child Health and Human Development Neonatal Research Network, Neurodevelopmental and growth impairment among extremely low-birth-weight infants with neonatal infection. JAMA. (2004) 292:2357–65. 10.1001/jama.292.19.2357 15547163

[4] Centers for Disease Control Prevention. Ventilator-associated pneumonia (VAP) event, July 2013 CDC/NHSN protocol corrections, clarification and additions. (2013). Available online at: http://www.cdc.gov/nhsn/PDFs/pscManual/6pscVAPcurrent.pdf (accessed February 28, 2022).

[5] Garner JS Jarvis WR Emori TG Horan TC Hughes JM. CDC definitions for nosocomial infections 1988. Z Arztl Fortbild. (1991) 85:818–27. 10.1016/0196-6553(88)90053-3 1659046

[6] Krankenhaus Infektions Surveillance System:Protokoll. Surveillancenosokomialer Infektionen bei Frühgeborenen mit einem Geburts-gewicht<1.500 g (NEO-KISS). (2009). Available online at: http://www.nrzhygiene.de/fileadmin/nrz/download/NEOKISSProtokoll221209.pdf.8 (accessed February 28, 2022).

[7] Foglia E Meier MD Elward A. Ventilator-associated pneumonia in neonatal and pediatric intensive care unit patients. Clin Microbiol Rev. (2007) 20:409e25. 10.1128/CMR.00041-06 17630332 PMC1932752

[8] Goldsmith JP Edward HK. Assisted Ventilation of the Neonate. Chapter 24, 5th ed. Saunders: Elsevier (2011), p, 426–35.

[9] ECDC SURVEILLANCE REPORT. Point prevalence survey of healthcare-associated infections and antimicrobial use in European acute care hospitals 2011–2012. Available online at: https://www.ecdc.europa.eu/sites/default/files/media/en/publications/Publications/healthcare-associated-infections-antimicrobial-use-PPS.pdf (accessed March 3, 2022).

[10] Gladilova A Ribarova N. Dynamics of prevalence and epidemiological characteristics of nosocomial infections in newborns in the period 2010-2011. Bulg Med J. (2012) 6:19–25 (In Bulgarian language) Available online at: http://cml.mu-sofia.bg:8080/jspui/handle/10861/467

[11] Ordinance N3 of 8.05.2013 on the approval of a medical standard for prevention and control of nosocomial infections. (2013). Available online at: https://www.mh.government.bg/media/filer_public/2015/11/18/prevenciq-control-vutrebolnichniinfekcii.pdf (In Bulgarian language). (accessed February 28, 2022).

[12] Yalaz M Altun-Koroglu O Ulusoy B Yildiz B Akisu M Vardar F. Evaluation of device-associated infections in a neonatal intensive care unit. Turk J Pediatr. (2012) 54:128–35. 22734298

[13] Mohammed D. El Seifl OS. Bacterial nosocomial infections in neonatal intensive care unit, Zagazig University Hospital, Egypt. Egypt Pediatr Assoc Gazette. (2014) 62:72–9. 10.1016/j.epag.2014.10.001

[14] Afjeh SA Sabzehei MK Karimi A Shiva F Shamshiri AR. Surveillance of ventilator-associated pneumonia in a neonatal intensive care unit: characteristics, risk factors, and outcome. Arch Iran Med. (2012) 15:567–71. 22924377

[15] Cernada M Aguar M Brugada M Gutiérrez A López JL Castell M. Ventilator-associated pneumonia in newborn infants diagnosed with an invasive bronchoalveolar lavage technique: a prospective observational study. Pediatr Crit Care Med. (2013) 14:55–61. 10.1097/PCC.0b013e318253ca31 22791095

[16] Aelami MH Lotfi M. Zingg W. Ventilator-associated pneumonia in neonates, infants and children. Children. (2014) 3:30. 10.1186/2047-2994-3-30

[17] Tan B Zhang F Zhang X Huang YL Gao YS Liu X. Risk factors for ventilator-associated pneumonia in the neonatal intensive care unit: a meta-analysis of observational studies. Eur J Pediatr. (2014) 73:427e34. 10.1007/s00431-014-2278-6 24522325

[18] Chastre J. Conference summary: ventilator-associated pneumonia. Respir Care. (2005) 50:975–83. Available online at: https://rc.rcjournal.com/content/50/7/975 15972117

[19] Stover BH Shulman ST Bratcher DF Brady MT Levine GL Jarvis WR. Nosocomial infection rates in US children's hospitals' neonatal and pediatric intensive care units. Am J Infect Control. (2001) 29:152–7. 10.1067/mic.2001.115407 11391276

[20] Almuneef M Memish ZA Balkhy HH Alalem H Abutaleb A. Ventilator-associated pneumonia in a pediatric intensive care unit in Saudi Arabia: a 30 month prospective surveillance. Infect Control Hosp Epidemiol. (2004) 25:753–8. 10.1086/502472 15484800

[21] Tripathi S Malik GK Jain A. Kohli N. Study of ventilator associated pneumonia in neonatal intensive care units: characteristics, risk factors and outcome. Internet J Med Uptodate. (2009) 5:12–9. 10.4314/ijmu.v5i1.49288

[22] Apisarnthanarak A Holzmann-Pazgal G Hamvas A Olsen MA Fraser VJ. Ventilator-associated pneumonia in extremely preterm neonates in a neonatal intensive care unit: characteristics, risk factors, and outcomes. Pediatrics. (2003) 112:1283e9. 10.1542/peds.112.6.1283 14654598

[23] Rodwell RL Leslie AL Tudehope DI. Early diagnosis of neonatal sepsis using a hematologic scoring system. J Pediatr. (1988) 112:764–9. 10.1016/S0022-3476(88)80699-1 3361389

[24] Cordero L Samuels P. Hillman T. Neutropenia in infants born to women with severe preeclampsia. Prenat Neonat Med. (1996) 1:363–70.

[25] Ng PC. The effectiveness and side effects of dexamethasone in preterm infants with bronchopulmonary dysplasia. Arch Dis Child. (1993) 68:330–6. 10.1136/adc.68.3_Spec_No.330 8466274 PMC1590377

[26] Badr MA Ali YF Albanna EA Beshir MR Amr GE. Ventilator associated pneumonia in critically-ill neonates admitted to neonatal intensive care unit, Zagazig university hospitals. Iran J Pediatr. (2011) 21:418–24. 23056825 PMC3446123

[27] El-Ward AM Warren DK. Fraser VJ. Ventilator associated pneumonia in pediatric intensive care unit: Risk factor and outcomes. Pediatrics. (2002) 109:758–64. 10.1542/peds.109.5.758 11986433

[28] Baltimore RS. The difficulty of diagnosing ventilator-associated pneumonia. Pediatrics. (2003) 112:1420–1. 10.1542/peds.112.6.1420 14654620

[29] Rangelova V Kevorkyan A Krasteva M Dermendzhiev T Kalchev Y. Five-year retrospective epidemiological study of nosocomial infections in the neonatal intensive care unit. Pediatrics Suppl. (2018) 57:47–51. (In Bulgarian language) Available online at: https://www.researchgate.net/publication/329060116_Petgodisno_retrospektivno_epidemiologicno_proucvane_na_nozokomialni_infekcii_v_neonatologicno_intenzivno_otdelenie26744145

[30] Gupta A Della-Latta P Todd B San Gabriel P Haas J Wu F. Outbreak of extended-spectrum beta-lactamase-producing *Klebsiella pneumoniae* in a neonatal intensive care unit linked to artificial nails. Infect Control Hosp Epidemiol. (2004) 25:210–5. 10.1086/502380 15061412

[31] Lee PL Lee WT Chen HL. Ventilator-associated pneumonia in low birth weight neonates at a neonatal intensive care unit: a retrospective observational study. Pediatr Neonatol. (2017) 58:16–21. 10.1016/j.pedneo.2015.10.014 27246111

[32] Faresjö T Faresjö A. To match or not to match in epidemiological studies–same outcome but less power. Int J Environ Res Public Health. (2001) 7:325–32. 10.3390/ijerph7010325 20195449 PMC2819792

[33] Eurostat. Population on 1 January 2021 by N 327 UTS 2 region. Available online at: https://ec.europa.eu/eurostat/databrowser/view/tgs00096/default/table?lang=en (accessed March 3, 2021).

